# Neural networks for faster laser ultrasound tomography in tissue phantoms

**DOI:** 10.1016/j.pacs.2026.100798

**Published:** 2026-01-13

**Authors:** Ahmed Al Fuwaires, Peter Lukacs, Don Pieris, Geo Davis, Helen Mulvana, Katherine Tant, Theodosia Stratoudaki

**Affiliations:** a Department of Electronic and Electrical Engineering, University of Strathclyde, 204 George st, Glasgow, G1 1XW, UK; b School of Engineering, University of Glasgow, University Avenue, Glasgow, G12 8QQ, UK

**Keywords:** Laser ultrasound, Biomedical imaging, Convolutional neural network, Image reconstruction, Speed of sound map

## Abstract

Speed of sound (SoS) mapping provides quantitative and localised information about a material’s acoustic properties, allowing identification of spatial compositional changes. In biomedical applications, SoS variations can inform tissue characterisation or improve image reconstruction algorithms that typically assume a constant SoS. However, conventional time-of-flight (ToF) tomography methods remain computationally intensive. This study presents experimentally derived tomographic reconstructions of SoS maps of heterogeneous structures from all-optically acquired data using a convolutional neural network (CNN). The CNN, trained on simulated data, enables near real-time, high-quality tomographic reconstructions. The novelty of this work lies in the integration of a laser ultrasound (LU) data acquisition setup with a CNN-based reconstruction approach, demonstrating its potential for remote and flexible inspection of biomedically relevant samples. The CNN was trained using simulated data based on ultrasonic wave propagation models and achieved tomographic reconstructions of a 77 mm × 77 mm area in less than 6 ms. Data were acquired from four tissue-mimicking phantoms (30 mm diameter) with inclusions of varying size (minimum 6 mm diameter) and SoS (minimum variation 25 m/s). When compared with published, in vivo studies using mammography (MM), conventional ultrasound, and magnetic resonance imaging (MRI), the proposed method yielded 5.73% mean sizing error for phantoms and inclusions relative to the ground truth, highlighting the clinical potential of the LU-CNN framework and the need for further in vivo testing. These findings underscore the method’s potential as a precise, faster alternative to conventional imaging and reconstruction methods used in clinical practice.

## Highlights

• Fully noncontact laser ultrasound tomographic setup for speed-of-sound (SoS) mapping.
• Rapid image reconstruction achieved by end-to-end convolutional neural network (CNN).
• CNN trained exclusively on simulated time-of flight (ToF) matrices and SoS maps.
• Experimental validation conducted on tissue-mimicking phantoms.
• Novel two-stage ToF picker framework improves travel-time estimation accuracy.

## Graphical abstract

**Figure:**
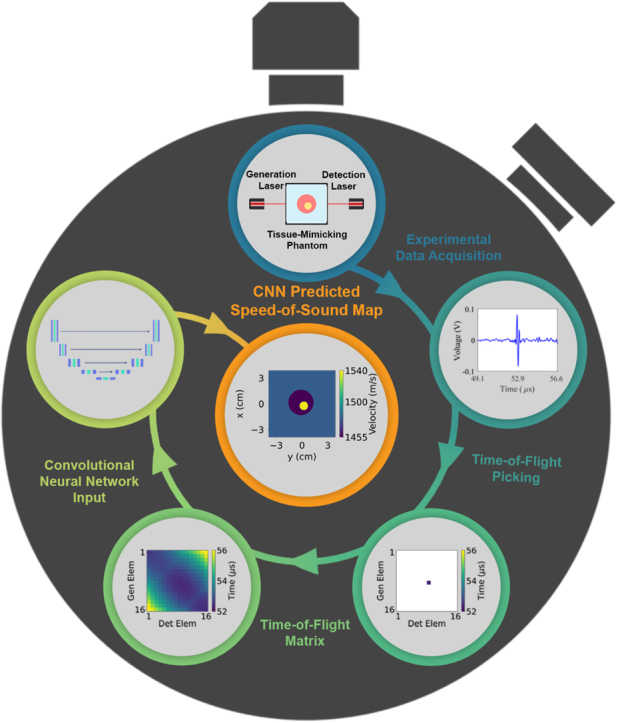

## 1 Introduction

Ultrasound computed tomography (UCT) is an imaging modality capable of providing insight into the acoustic properties of biological tissues within a region of interest, enabling reconstruction of detailed structural and compositional information. Unlike X-ray imaging and mammography (MM), UCT offers a non-invasive modality that avoids ionising radiation and may thus enable more frequent clinical examinations. It has also demonstrated comparable performance to magnetic resonance imaging (MRI) in visualising soft tissue lesions but with reduced acquisition times [1]. These advantages have driven substantial research interest in UCT, highlighting its potential in various clinical diagnostic contexts.

UCT can be used to reconstruct quantitative images of inhomogeneous media, such as tissue, providing spatial maps of material properties such as speed of sound (SoS) and acoustic attenuation (AA) [2]. This is typically achieved by surrounding the object of interest with a wide-angle array of transducers which both generate and detect ultrasound, and recording the ultrasonic wavefield for tomographic reconstruction. In biomedical applications, these maps can be used to distinguish between different types of tissue [3], [4], as well as for correcting aberrations caused by assumptions of homogeneous wave propagation [5], [6], [7].

Conventional ultrasound tomography using ultrasound transducers is a non-invasive technique and can produce high-resolution images. However, several factors limit its widespread clinical usage. Transducers generally have a limited acoustic bandwidth, reducing resolution and contrast [8], and signal generation may suffer from pulse-to-pulse fluctuations which can impact image consistency. Additionally, the widespread use of lead-based piezoelectric materials raises environmental and health-related concerns [9]. While lead-free materials have been proposed as safer substitutes, they are yet to offer the same level of performance as their lead-based alternatives. In addition, the inverse relationship between transducer size and sensitivity imposes further performance constraints. As transducer miniaturisation progresses, fabrication challenges limit element dimensions, consequently reducing operating frequency, pitch and aperture. These limitations ultimately reduce the accuracy of the reconstructed images.

Laser Ultrasound (LU) presents a promising alternative that addresses many of these constraints. As a non-contact method, LU uses lasers to generate ultrasound through the photoacoustic effect, thereby eliminating the need for physical coupling. For applications where skin integrity is compromised, such as burns, open wounds or infections and direct contact holds the risk of causing tissue damage or routes for infection entry, LU presents a unique method of gathering clinically relevant imaging data that may otherwise be unavailable [10]. Furthermore, it can facilitate imaging in challenging or difficult to reach locations, including those only accessible via minimally invasive delivery methods such as optical fibre coupling [11], [12]. LU enables a high level of control and flexibility over ultrasonic array design parameters, including the number of elements and their configuration, allowing customisation for specific imaging requirements [13]. Additionally, its broadband signal generation, which can be in the order of >30 MHz [14], offers significant advantages in biomedical imaging compared to the limited bandwidth associated with conventional ultrasound transducers (generally limited to a few MHz). This is particularly beneficial in media with frequency-dependent attenuation and variations in speed of sound (SoS), where broadband signals enhance imaging performance [15]. LU signals also exhibit shorter ultrasonic pulse widths of <40 µs as well as decreased reverberations [14], [16] which can improve time-of flight (ToF) picking accuracy, which is critical for tomographic reconstruction [17]. As a result of the non-contact and broadband capabilities described, LU and photoacoustics (PA) have generated significant interest within biomedical imaging [18].

Optical methods for tomographic imaging, including LU and PA, have gained significant attention in biomedical applications [18], [19], [20]. LU ToF tomography has previously been demonstrated using optical absorbers designed to generate high-amplitude ultrasonic pulses. Unlike PA, LU aims to reduce the optical absorption depth in the irradiated medium to generate high amplitude ultrasonic pulses. This is typically achieved by strongly absorbing the laser radiation, resulting in a point ultrasonic source, while minimising the amount of radiation absorbed by the tissue. Several studies have explored this technique. For example, in [21], [22], pulsed lasers were directed onto carbon fibre and thin wire absorbers respectively, to produce ultrasonic pulses. More recently, engineered absorbers have been developed to customise key ultrasonic parameters including bandwidth, pressure and angle of divergence [23]. A hybrid setup with LU transmission and piezoelectric detection was used in [24] to produce both SoS maps and reflection images of tissue phantom. In these examples of LU for ToF tomography, the systems employ conventional piezoelectric transducers for signal detection, which may limit sensitivity and bandwidth, and have large array element size compared to a point detector. Optical detection of ultrasound would offer similar advantages to LU generation — broadband ultrasonic detection and flexibility of array configuration — as shown in PA tomography systems [25].

Conventional ultrasound tomography reconstruction methods can be divided into waveform-based methods and ray-based methods. Waveform-based methods involve solving the acoustic wave equation by modelling the propagating wave and, therefore, take into account factors such as diffraction and scattering [26], [27], [28]. In ray-based methods, the principles of geometric acoustics are used in order to reconstruct SoS maps by utilising the ToF of signals from transmitter to receiver and assigning them to integrals along the ultrasound propagation path [29], [30]. Traditionally, both approaches are iterative, where an initial estimate of the SoS map is created and iteratively perturbed until the misfit with the experimentally observed data is minimised [31]. This process is computationally intensive, with reconstruction times ranging from minutes to hours, limiting real-time imaging and characterisation capabilities [30], [32], [33].

Recently, deep learning (DL) has shown potential in biomedical imaging, particularly in generating tomographic images directly from raw signals [34], [35], [36] or ToF data [37], [38]. The present study uses ToF inputs which shift in response to SoS variations. CNN-based reconstructions using ToF inputs have demonstrated improved accuracy over raw signal inputs [39], with low mean squared error (MSE) values and high structural similarity index (SSIM) scores when compared with various conventional reconstruction algorithms [40]. ToF approaches also reduces data size and computational complexity, enabling faster image reconstruction, which in turn creates the possibility of real-time monitoring [40], [41]. However, effective use of ToF for image reconstruction requires accurate ToF estimation. Many ToF picking algorithms rely on signals with a wide frequency bandwidth, but conventional piezoelectric transducers often produce narrowband signals, increasing the likelihood of inaccurate ToF estimation [17], [42], [43].

This paper demonstrates the feasibility of producing tomographic reconstructions of SoS maps of heterogeneous structures from LU data in near real-time using CNNs. By leveraging deep learning, we aim to radically reduce reconstruction times, overcoming the computational bottleneck of traditional iterative methods. The novelty of this work lies not only in the integration of LU with CNN-based reconstruction but also in showcasing LU’s potential for remote, flexible inspection of biomedically relevant samples, as validated through tissue-mimicking phantoms.

**Fig. 1:**
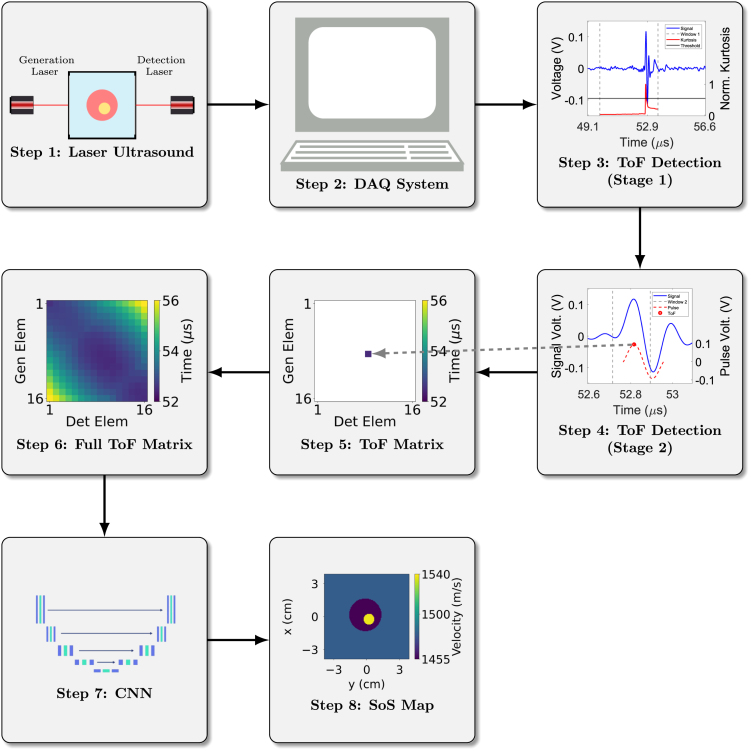
Methodology for data acquisition and image reconstruction. Ultrasonic signals are acquired from the immersion tank using the generation and detection lasers. The signals are collected on the lab PC which process the signals through the two-stage ToF picker method to select the ToF. The ToF values populate the ToF matrix and are subsequently used as the input to a trained CNN which reconstructs the SoS map of the immersion tank.

The overall methodology is illustrated in Fig. 1. The rest of the paper is organised as follows: Sections 2.1, 2.2 outline the creation of simulated training data. Section 2.3 presents the developed CNN architecture and associated parameters. Section 2.4 gives the two-stage method utilised to pick the ToF for each experimentally acquired signal. Section 3 illustrates the LU experimental setup and the phantom mimicking materials investigated. Section 4 presents experimental results and comparison with ground truth, followed by discussion in Section 5 and conclusions in Section 6.

## 2 Methodology

To enable real-time image reconstruction using CNNs, the generation of a large and diverse training dataset was necessary. Relying solely on experimentally data imposes limitations on both size and variability in the training set, as acquisition is often time-intensive and resource-demanding. To address this constraint, the Anisotropic Locally Interpolated Fast Marching Method (ALI-FMM) [44] was employed as a scalable and efficient method for synthesising simulated ToF matrices from SoS maps for training the CNN. In order to further reduce image reconstruction time, an automated ToF picker was developed to correctly identify the ToF for the experimentally acquired signals.

### 2.1 Anisotropic Locally Interpolated Fast Marching Method (ALI-FMM)

**Fig. 2:**
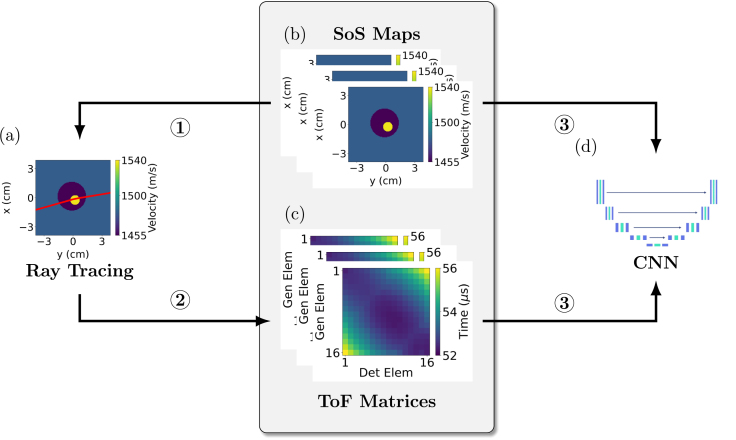
Flow diagram illustrating CNN training using the ALI-FMM algorithm on simulated SoS maps to create pairs of ToF matrices. (1) Ray tracing is performed on simulated SoS maps (a). (2) The ray tracing code creates pairs of ToF matrices for each corresponding SoS map. (3) The resulting pairs of SoS maps (b) and ToF matrices (c) are used to train the CNN (d). In (a) and (b), the x label represents the laser generation and detection scanning axis while the y label corresponds to the signal propagation path through the immersion tank. In (c), the x and y axis indices correspond to the detection and generation elements respectively.

A major challenge to implementing data driven DL frameworks is the scarcity of diverse and high-quality labelled data, which is essential for model training. To address this constraint, synthetic travel time data were generated by a computationally efficient ray-based model as described in [44]. The Anisotropic Locally Interpolated Fast Marching Method (ALI-FMM) extends the classical Fast Marching Method (FMM) [45] to consider heterogeneous and locally anisotropic media. Although the present study is limited to isotropic materials, use of this model will facilitate extension to anisotropic media in the future with no increase in computational time. The input to the ALI-FMM algorithm is a matrix of the material properties of interest (in this case, a SoS map) at all points on a grid (in the present study 128 × 128 pixels). The algorithm then models wave-front propagation through the medium from a given source point to generate a travel-time field which estimates the shortest travel time between the source and every point in the grid. The travel-time field is interpolated onto a finer grid (640 × 640 pixels) and rays are then traced between the known locations of the generation and detection points to produce a more accurate approximation of the true travel time. ToF matrices can then be constructed by modelling the shortest travel-time between every pair of known generation detection points in this way, and paired with their corresponding SoS maps, these serve as the input and output training pairs for the CNN. A process flow diagram is shown in Fig. 2.

### 2.2 Synthetic training data

To construct the synthetic training data for this study, 20,000 SoS maps of size 128 × 128 pixels were generated to serve as the input for the ALI-FMM algorithm described in Section 2.1. In order to ensure training data variety facilitating the development of a robust CNN, SoS maps were randomly generated using the parameters outlined in Table 1. Parameters specified by ranges or discrete sets were sampled uniformly at random to ensure unbiased parameter selection and diversity in the training dataset. To further enhance variability in the training dataset, two categories of phantom SoS maps were generated in equal proportion (50% each): one consisting of a single inclusion and another consisting of two inclusions. The centre of the phantom was randomly shifted axially and laterally with reference to the centre of the SoS map (the ‘offset centre shift’), the range of values of which are given in Table 1. Following selection of the phantom location, its diameter was selected at random from the specified range. In phantoms containing two inclusions, the minimum diameter of the phantom was increased to accommodate both inclusions. The location of the inclusion(s) was randomly chosen but constrained to ensure the entire volume of each inclusion lay fully within the boundaries of the phantom. Finally, the material of each of the inclusions was then randomly assigned to be either water or 10 wt% Glycerol, with respective SoS 1480 m/s or 1538 m/s.

The ALI-FMM was used to calculate travel times between all combinations of generation and detection element locations (determined by the experimental setup described in 3.1) through each simulated SoS map. As shown in [46], [47], [48], increasing the number of projection angles has been shown to enhance imaging accuracy ultrasonic tomography experiments. Therefore, for each SoS map, the ALI-FMM code was executed twice for each view: once with the phantom positioned at 0° and then rotated at 90° about its longitudinal axis. The resulting ToF data are denoted as $T_{1}$ for the initial acquisition and $T_{2}$ for the rotated acquisition. The entire ToF dataset was then normalised to unity to facilitate input to the CNN. The pairs of ToF matrices, one 16 × 16 matrix corresponding to each SoS map, were concatenated into a 32 × 32 matrix to facilitate the CNN’s requirement for a 2D matrix input in the form $\left(\begin{array}{cc} T_{1} & 0\\ 0 & T_{2} \end{array}\right)$, where 0 represents a 16 × 16 zero matrix. This structure enables integration of two separate single-channel datasets in a 2D dual channel input. Such dual channel representations have been shown to improve model accuracy when compared multiple single-channel structures [49]. The zero-padding block-diagonal representation serves to isolate the independent matrices, $T_{1}$ and $T_{2}$, such that feature extraction in the combined matrix is localised within the original matrices optimising model performance [50], [51]. Additionally, the SoS map data was normalised to lie within the range [0, 1] by assigning the integer 0 to gel, 0.5 to water and 1 to 10 wt% Glycerol. This data scaling step is critical for model convergence stability and ensuring optimal performance of the CNN [52].

**Table 1:** The parameters utilised for the automated training dataset creation.

Training Data Parameter	Value (m/s)	Range (mm)	Increment (mm)
Background Velocity	1480		
Phantom Velocity	1455		
Inclusion Velocity	1480 or 1538		
Offset Centre Shift (lateral and axial)		−1.2–1.2	0.6
Phantom Diameter (1 Inclusion)		9.7–29.6	0.6
Inclusion Diameter (1 Inclusion)		4.8–11.5	0.6
Phantom Diameter (2 Inclusions)		23.6–29.6	0.6
Inclusion Diameter (2 Inclusions)		4.8–11.5	0.6

### 2.3 Network architecture

To achieve real-time image construction, the CNN architecture, referred to as mWnet_4, described in [36] was adapted and implemented in TensorFlow [53]. The modified CNN model is the one employed in step 7 of Fig. 1. This model begins with feature extraction using convolutional layers with a stride of 2 to down-scale the feature map, followed by residual blocks for encoding. The domain transform is handled by high-level layers with large receptive fields, enhanced by multiple down- and up-scaling (DUS) units inspired by image denoising and super-resolution techniques [54], [55], [56]. This approach efficiently increases the number of hidden layers, providing a large receptive and projective field that encompasses all elements in the inputs and outputs. This means that the network can capture detailed information from a broad area of the input sensor data, ensuring that even subtle features are recognised and integrated into the final image. Finally, the reconstruction phase uses residual blocks and depth to space with a block size of 2 convolutional layers to up-scale features, with skip connections to reuse extracted features for accurate reconstruction. A SoS map is then reconstructed through a single forward pass of the trained network using input ToF data, as outlined in steps 6–8 of Fig. 1. This ToF-CNN reconstruction approach is significantly faster than traditional iterative tomography methods and as a result, the network can transform ultrasound sensor data into high-quality images quickly and accurately, enhancing real-time imaging capabilities.

In contrast to the architecture proposed in [36], which uses frequency-dependent sound pressure data as the input, this study utilises ToF data. The input tensor is the concatenated ToF matrix of size 32 × 32, as detailed in Section 2.2 This matrix is subsequently zero-padded to match the output SoS map size of 128 × 128 pixels. The implementation used in this work utilises 4 DUS blocks to further enhance reconstruction performance. The implementation in [36] utilises the sub-pixel layers for up-scaling, however, as this not available in TensorFlow, this study substitutes them with a depth-to-space with a block of size 2. The Adam optimiser [57] was used for training with a learning rate of 10^−4^ and a mean-square error (MSE) loss function. To improve model performance, the learning rate was adaptively reduced by a factor of 0.5 if no improvement in validation performance was observed to the model over 8 consecutive epochs. The convolutional units were followed by a rectified linear unit (ReLu) activation function. The model was trained on a batch size of 16 for 100 epochs similar to the implementation used in [35]. Of the 20,000 simulated datasets, 17,820 were used for training, 200 including the 4 experimental datasets were used for testing and 1980 used for validation of the model. It took approximately 10 h and 15 min to train the network on the lab PC (64-bit operating system, x64-based processor, 64 GB RAM, NVIDIA GeForce RTX 3090) with a total number of parameters of approximately 115.3 million.

### 2.4 Automated Time-of-Flight picking method

In order to automate the selection of the ToF for each experimentally acquired signal, a two-stage ToF picking method was developed, corresponding to steps 3–6 of the workflow shown in Fig. 1. The implementation of the two-stage ToF picker demonstrated enhanced accuracy compared to single-stage picking approaches, as detailed in Section 5. The two key stages of the picker method are presented Fig. 3. Note that, prior to the picking method, each signal is bandpass filtered between 1 and 7 MHz to enhance the signal-to-noise ratio (SNR) of the ultrasonic data by suppressing unwanted frequencies while still preserving the signal onset [58]. A unique time window is applied to the filtered signal in Stage 1 (Fig. 3(a)). This window is defined by the minimum and maximum transmission times estimated using the distances between the generation and detection points, as well as the highest and lowest velocities of interest. Thus, prior knowledge of the experimental geometry and the ultrasonic velocities present in the propagating medium are required for this step. In this research, these velocities are chosen to reflect a range typically encountered in biomedical applications, between 1455 m/s and 1538 m/s. Once this window is created, the kurtosis in the windowed signal is analysed in a sliding time window. Kurtosis is a dimensionless, statistical metric characterising the tailedness and peakedness of a given distribution, quantifying deviations from Gaussianity [59]. Considering that the pre-triggered signal is a stationary process, the mean and variance are time-invariant and higher order statistics are expected to be constant. The introduction of a pulse signal belonging to a non-Gaussian distribution is expected to cause an increase in kurtosis, which is given by [60], [61]

(1) $$\kappa \left(k\right)=\frac{\frac{1}{N}\overset{N-1}{\underset{i=0}{\sum }}{\left[s\left(k+i\right)-\overset{¯}{s}\right]}^{4}}{{\left(\frac{1}{N}\overset{N-1}{\underset{i=0}{\sum }}{\left[s\left(k+i\right)-\overset{¯}{s}\right]}^{2}\right)}^{2}},$$

where N is the number of samples in the sliding window, s(k) is the signal, $\overset{¯}{s}$ is the mean of the windowed samples and κ(k) is the kurtosis at index k. Subsequently, a threshold is defined with the first peak that surpasses this being selected as the onset of the pulse. It was found empirically that a window size of 2 µs and a threshold of 55% the maximum value of calculated kurtosis yielded the highest performance in pulse onset identification. The sample number k at which this peak occurs is then used to define a smaller window. The smaller window was of size 0.18 µs, centred around sample k, with 0.08 µs preceding and 0.1 µs following sample k. The second stage of the picking process (Fig. 3(b)) uses the cross-correlation method to extract the ToF [62] within the newly created window. In this approach, the detected signal is cross-correlated with a reference transmitted signal (in the presented experiments, the reference ultrasonic signal is acquired in the presence of water only, in the absence of phantom, see Section 3.1), resulting in a cross-correlation sequence defined as

(2) $$R\left(m\right)=\overset{L}{\underset{j=1}{\sum }}x\left(j\right)y\left(m+j\right),$$

where x is the signal detected at the receiver, y is the reference transmitted signal, L is the length of x and R(m) is the cross-correlation value at lag point m. The sliding inner product is performed and the lag value at which R(m) reaches the maximum value indicates the point of highest similarity. This lag value is utilised to select the ToF which is then recorded in the ToF matrix as shown in Fig. 1.

Fig. 3The stages involved in picking the ToF of the signal. (a) An initial window is created which is defined based on the SoS within the medium and the distance between the generation and detection points, indicated by the dashed grey lines. The signal’s kurtosis is plotted in red with the black line representing the kurtosis threshold used for peak selection. (b) A narrower window is applied around the peak kurtosis region, shown by the dashed grey lines. Cross-correlation is performed on the detected signal in blue and reference pulse signal shown in the dashed red line with marked lag point m where R(m) is maximum Eq. (2)).Fig. 3

**(a):**
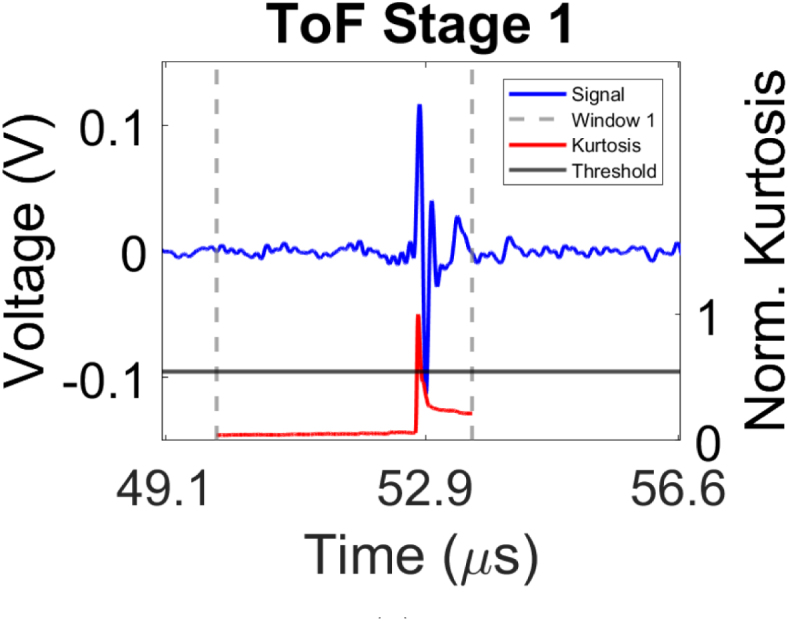
.

**(b):**
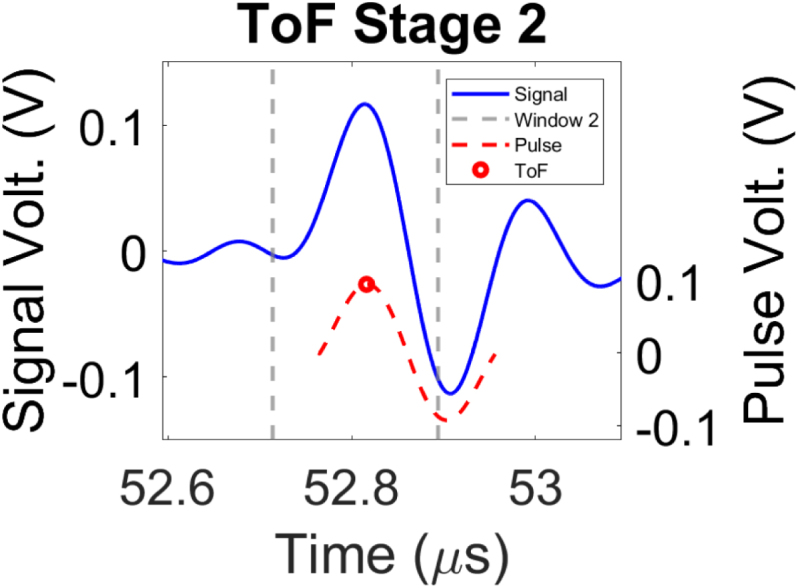
.

## 3 Experimental setup and materials

### 3.1 Experimental setup

**Fig. 4:**
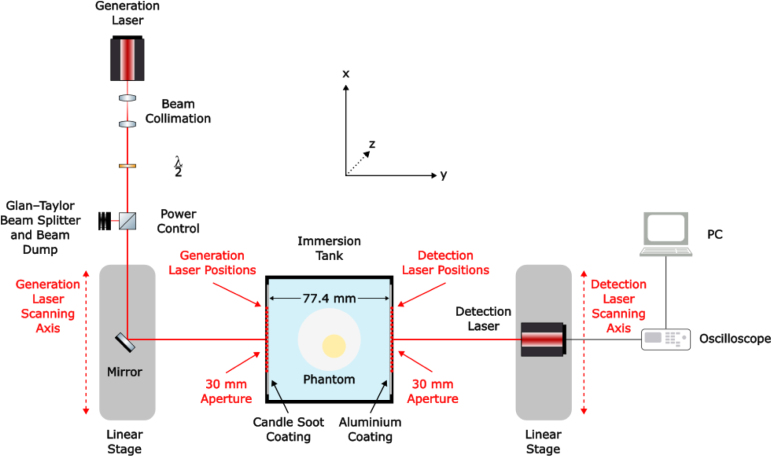
A schematic of the experimental setup used for data acquisition (top view). The immersion tank is shown with a phantom.

A schematic of the experimental setup used in this work is presented in Fig. 4. The laser ultrasound setup was constructed to generate and detect ultrasonic pulses through a medium containing various phantoms. Acoustic waves at the surface of the immersion tank were excited using a pulsed Q-switched Nd:YAG IR laser (NL204, Ekspla) operating at 1064 nm, with a 10 ns full width at half maximum (FWHM) pulse width and a 1 kHz repetition rate. The tank measured approximately 77.4 mm ×77.4 mm with a height of 60 mm, corresponding to a volume of ∼0.4 L. The incident laser energy was 0.45 mJ per pulse and a cylindrical lens was used to focus the laser beam into a line with a length of 3.02 mm and a width of 1.05 mm on the surface of the immersion tank. The line source was oriented parallel to the z-axis to generate a cylindrically diverging wave in the x–y plane, the plane of reconstruction [63]. For detection, a continuous-wave (CW) HeNe laser (wavelength 633 nm) was employed as part of a laser Doppler vibrometer (VibroFlex Compact, Polytec), with approximate laser spot size diameter of 40 µm.

Data were acquired using a 16-element ultrasonic generation array and a 16-element ultrasonic detection array, synthesised by scanning the generation and detection lasers on opposite sides of the immersion tank. Both arrays had equally spaced elements, which were superimposed at each location in a through-transmission configuration. The aperture of the array was 30 mm, yielding a pitch of 2 mm. The distance between the generation and detection plates was fixed at 77.4 mm. Two datasets ($T_{1}$ and $T_{2}$) were acquired, corresponding to two viewing angles: one with the phantom positioned at 0° and another rotated 90° about its longitudinal axis within the immersion tank, consistent with the two-view synthetic datasets generated using the ALI-FMM algorithm (Section 2.2).

An optical absorber made of candle soot nanoparticles-polydimethylsiloxane (CSNPs-PDMS) was used in order to absorb the light of the ultrasonic generation laser and generate the ultrasonic pulse. This material was chosen due to its high energy conversion efficiency, reported as $4.41\times 10^{-3}$, which exceeds other commonly used composites including carbon nano-fibre ($1.66\times 10^{-3}$), carbon black ($0.34\times 10^{-3}$), gold nanoparticles ($0.18\times 10^{-3}$) and carbon nanotube ($0.18\times 10^{-3}$) [64]. The protocol for the creation and application of CSNP-PDMS coating on the glass plate (WG12012, Thorlabs) was based on a combined fabrication method presented in [14], [65] and is detailed in Appendix A. The detection plate was an aluminium coated mirror (PF20-03-F01, Thorlabs) to allow for high reflectivity of the detection laser. Both the CSNPs-PDMS and the reflective aluminium layer were in contact with the water in the immersion tank. The raw signals were acquired using the oscilloscope via a custom designed Python script for processing on the PC.

The system was first tested without a phantom to characterise the directivity of the laser source at the detection plate to understand the spatial propagation of the generated acoustic field. The amplitude of the laser pulse first arrival was plotted as a function of lateral distance from the centre point of the source. This approach allowed for quantification of the angular spread of the generated wave. A receive array aperture of 40 mm, comprising 41 detection points with 1 mm spacing was employed. The resulting directivity profile is presented in Fig. 5, revealing the divergent nature of the generated ultrasonic wave.

To assess the frequency spectrum of the generated pulse, the Fast Fourier Transform (FFT) of a time domain signal detected at the centre point of the laser source at the detection plate was performed. The analysis indicated a -6 dB ultrasound bandwidth ranging from 1.94 MHz to 11.98 MHz, with a frequency peak at 6.56 MHz (Fig. 6). The broadband frequency response increases the accuracy of the ToF picker method employed in this study. The spectral characteristics of this generated signal is primarily attributed to several parameters including the laser pulse duration, concentration of CS to PDMS and thickness of the coating [66]. Optimisation of these factors can be further tailored to meet application-specific imaging requirements.Fig. 5Normalised directivity profile of the generated ultrasonic signal. (a) Experimental setup illustrating the fixed generation laser at x=0mm while laterally scanning the detection laser across the linear scanning axis for an aperture size of 40 mm centred about x=0. (b) Amplitude of first arrival as a function of lateral position about x=0. The dashed grey lines indicate the 30 mm aperture employed in this study.Fig. 5

**(a):**
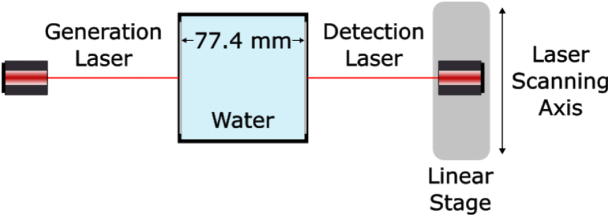
.

**(b):**
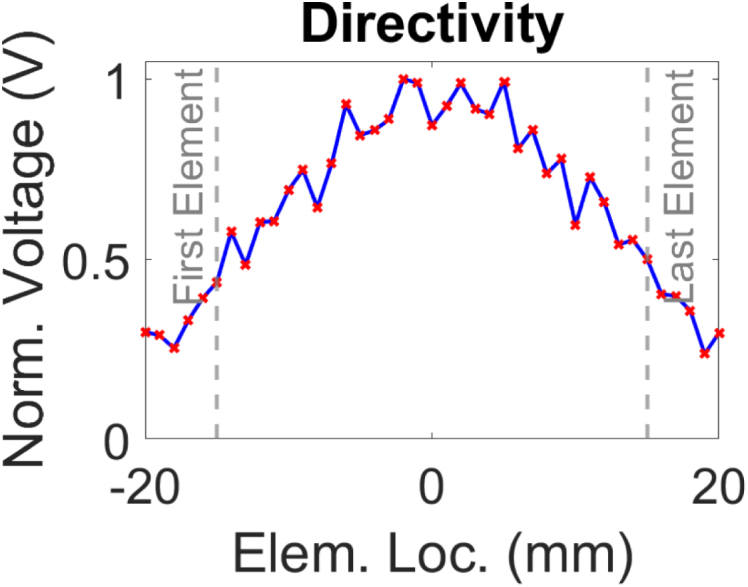
.

Fig. 6(a) Measured acoustic signal in the time domain and (b) the corresponding power spectrum normalised to 0 dB. The dashed grey line describes the bandwidth at -6 dB. The two black lines show the frequency range.Fig. 6

**(a):**
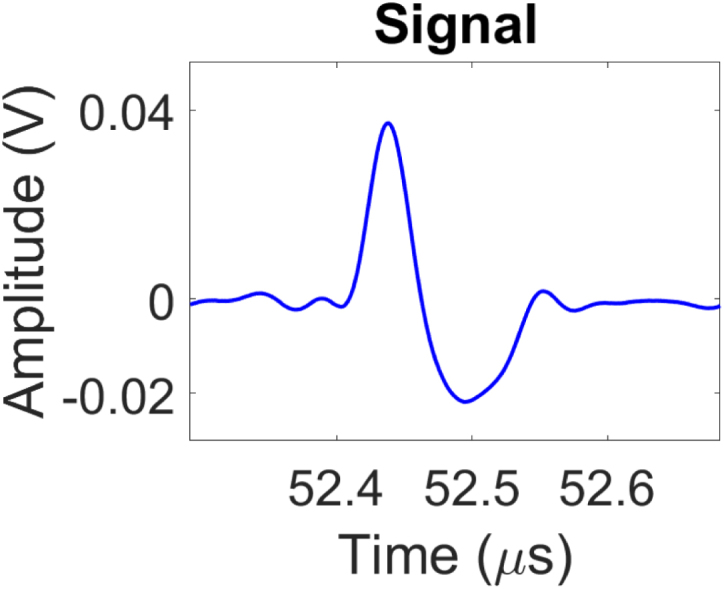
.

**(b):**
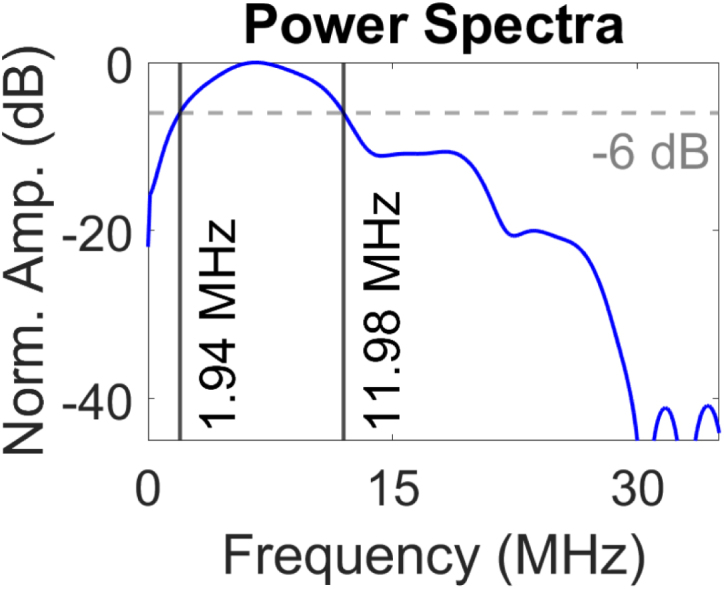
.

### 3.2 Phantom materials and fabrication

**Fig. 7:**
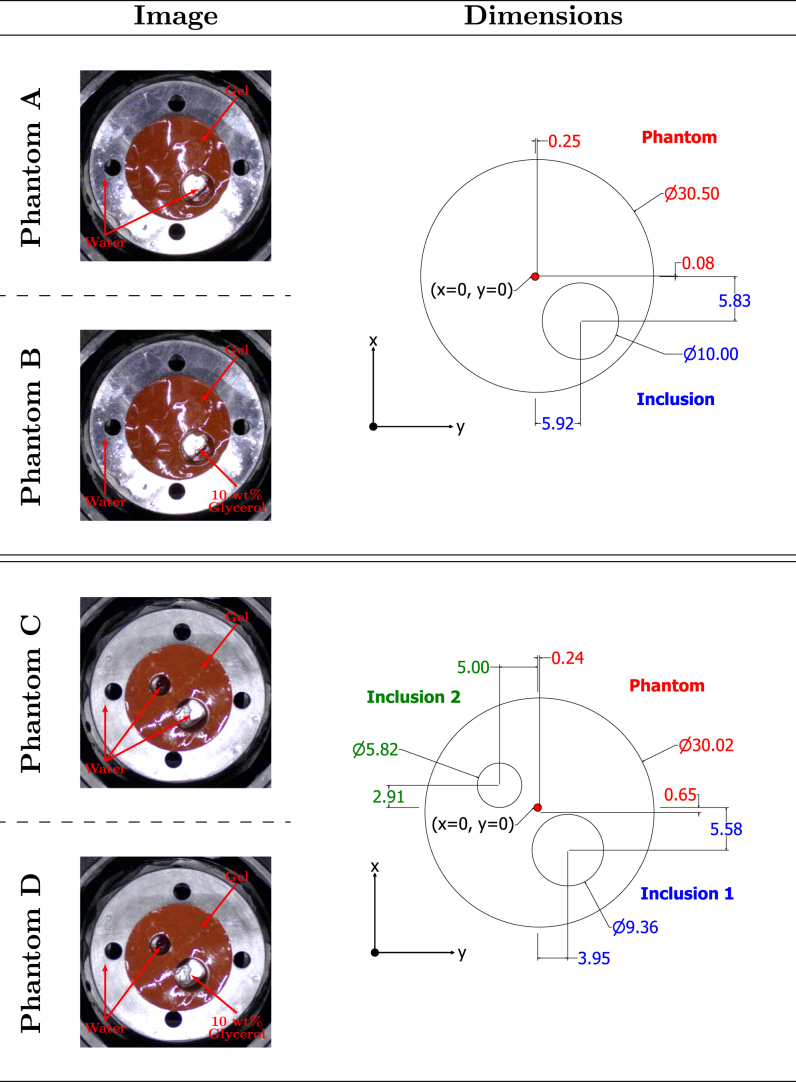
(left column): Top-view photos (ground truth) of the fabricated phantoms along with the contents of each. (right column): Schematic showing the dimensions of the corresponding phantoms and inclusions with respect to the centre of the immersion tank (shown by the red dot).

Two different tissue-mimicking phantoms were fabricated using the same gelatin-based material. The factors considered for the phantom design and material were acoustic similarity of phantom material to human tissue, design flexibility and ease of fabrication. The material selected was a tissue-mimicking gelatin (Gelatin #4, Humimic Medical) with acoustic properties within the range typical for human soft tissue (1420 m/s – 1680 m/s), particularly breast tissue, as outlined in Table 3. The phantoms were cylindrical and included one or two cylindrical inclusions. These inclusions, with varying SoS, were arranged to simulate a heterogeneous acoustic environment. Detailed specifications are provided in Table 2 and Fig. 7. The combination of phantom and inclusion fillers allowed for the four different cases for tomographic imaging and are referred to as Phantoms A, B, C and D.

In total, three materials were used in the experiments (SoS shown in Table 3): gel, deionised water and a mixture of deionised water and 10 wt% glycerol (8.18709, Sigma Aldrich) for an increased SoS [67]. Deionised water was also used to fill the tank. The smallest SoS variation among these materials was 25 m/s, comparable to the variation observed across different human tissues as shown in Table 3.

**Table 2:** Phantoms used and their corresponding diameters (Dia.), height, number of inclusions (No. of Inc.) and the contents of them.

Phantom A	Dia. (mm)	Height (mm)	No. of Inc.	
	30.50	30	1	
	Inc. 1 Dia. (mm)	Inc. 1 Content	Inc. 2 Dia. (mm)	Inc. 2 Content
	10.00	Water	x	x
Phantom B	Dia. (mm)	Height (mm)	No. of Inc.	
	30.50	30	1	
	Inc. 1 Dia. (mm)	Inc. 1 Content	Inc. 2 Dia. (mm)	Inc. 2 Content
	10.00	10 wt% Glycerol	x	x

Phantom C	Dia. (mm)	Height (mm)	No. of Inc.	
	30.02	30	2	
	Inc. 1 Dia. (mm)	Inc. 1 Content	Inc. 2 Dia. (mm)	Inc. 2 Content
	9.36	Water	5.82	Water

Phantom D	Dia. (mm)	Height (mm)	No. of Inc.	
	30.02	30	2	
	Inc. 1 Dia. (mm)	Inc. 1 Content	Inc. 2 Dia. (mm)	Inc. 2 Content
	9.36	10 wt% Glycerol	5.82	Water

The phantoms were fabricated on a custom-designed metallic base that attached to a rotation platform to interface the components, allowing for simple replacement of different phantom structures. The phantom fabrication protocol and an illustration of the components are provided in Appendix A, Appendix C. In order to obtain the ground truth of the phantoms, a camera was positioned above the immersion tank, which captured the top view of the phantom in the tank (viewing angle along the z axis shown in Fig. 4, with the captured images displayed in Fig. 7).

**Table 3:** Speeds of sound of materials used for phantoms and typical values reported for various breast tissues [68], [69], [70].

Phantom		Breast	
Material	SoS (m/s)	Tissue Type	SoS (m/s)
Water	1480	Fat	1420
Humimic Gel	1455	Glandular	1515
10 wt% Glycerol	1538	Breast	1510
		Skin	1680
		Small Lesion	1530
		Large Lesion	1560

## 4 Results

**Fig. 8:**
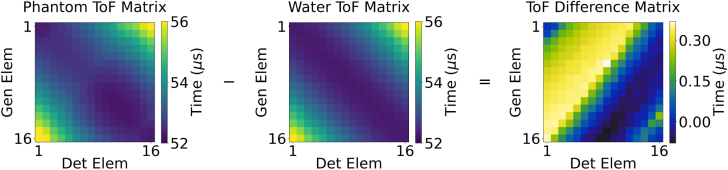
Example data illustrating a ToF matrix for a tissue phantom, water and the their corresponding ToF difference matrix.

To assess the accuracy of this tomographic methodology, data were acquired from the four phantoms (Table 2) using the experimental system described in Section 3.1. In order to visualise the difference between the resulting ToF matrices and to facilitate easier identification of acoustic propagation characteristics, a ToF difference matrix was constructed by subtracting the ToF matrix acquired from the phantom from the ToF matrix acquired from water only (Fig. 8). ToF difference matrices will be depicted throughout the remainder of this results section. It is important to note that the ToF matrix, and not the ToF difference matrix, served as the input to the CNN.

**Fig. 9:**
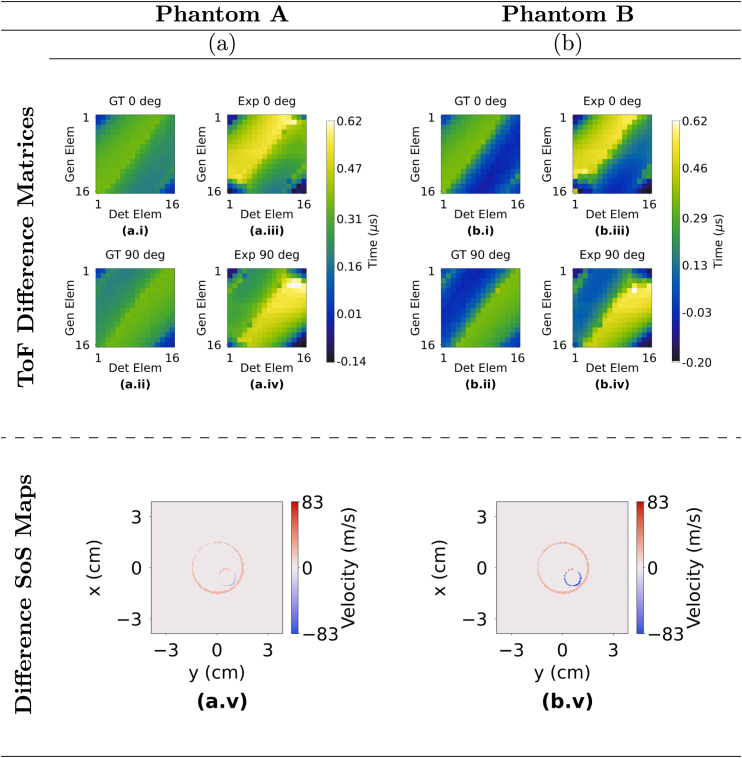
Sub-figures labelled a and b correspond to Phantoms A and B respectively. (a.i) and (b.i) ground truth $T_{1}$ matrices (GT 0°); (a.ii) and (b.ii) ground truth $T_{2}$ matrices (GT 90°); (a.iii) and (b.iii) experimentally acquired $T_{1}$ matrices (Exp 0°); (a.iv) and (b.iv) experimentally acquired $T_{2}$ matrices (Exp 90°); (a.v) and (b.v) are the difference SoS maps which show the difference between the CNN predicted and ground truth SoS maps for Phantoms A and B.

**Fig. 10:**
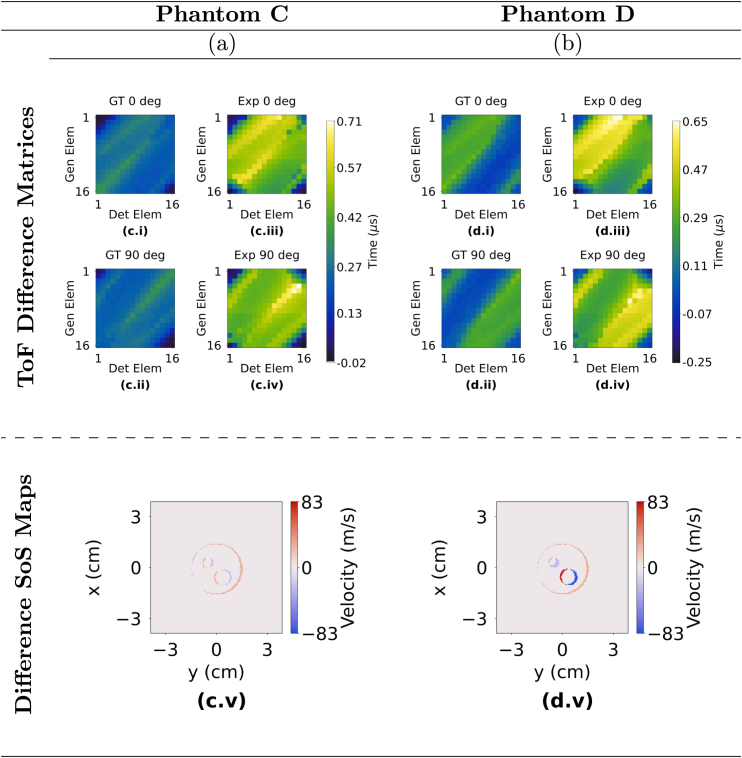
Sub-figures labelled c and d correspond to Phantoms C and D respectively. (c.i) and (d.i) ground truth $T_{1}$ matrices (GT 0°); (c.ii) and (d.ii) ground truth $T_{2}$ matrices (GT 90°); (c.iii) and (d.iii) experimentally acquired $T_{1}$ matrices (Exp 0°); (c.iv) and (d.iv) experimentally acquired $T_{2}$ matrices (Exp 90°); (c.v) and (d.v) are the difference SoS maps which show the difference between the CNN predicted and ground truth SoS maps for Phantoms C and D.

In order to validate the data, the experimentally acquired ToF difference matrices were compared against the ground truth data generated through simulations created using the ALI-FMM algorithm. Fig. 9, Fig. 10 show the ground truth and experimentally acquired difference matrices corresponding to the $T_{1}$ and $T_{2}$ views of the phantom geometry. While the ground truth and experimental difference matrices may appear visually distinct at first glance, the maximum difference across these matrices is only 0.5180 µs — approximately 1% of the input ToFs — which falls well within the CNN’s tolerance.

In order to visualise differences in the size and location of the phantoms and inclusions between both the ground truth and CNN predicted SoS maps, a difference SoS map was generated. The difference SoS map was computed by subtracting the ground truth SoS map from the corresponding CNN predicted SoS map. The resulting difference SoS maps for phantom A–D are also presented in Fig. 9, Fig. 10 and illustrate the accuracy of the CNN-predicted image.

The diameters and centres of the phantom and inclusions were measured and compared to ground truth measurements D in order to evaluate the accuracy of the CNN in reconstructing SoS maps. Diameters were determined by counting the number of pixels along the longest axis of the phantoms and inclusions. The midpoint of the measured diameter was identified as the centre and its location was referenced with respect to the centre of the immersion tank. The percentage change in diameter and spatial centre offset between the reconstructed and the ground truth images are depicted in Fig. 11 offering a clear depiction of the key values measured in Appendix D.

Two metrics were evaluated to quantify the magnitude of the differences between the CNN predicted and ground truth SoS maps: change in diameter and the positional offset of their geometric centres. The results presented in Appendix D indicate that the mean change in diameter between the ground truth and the CNN predicted image for the phantom, averaged across all four phantom cases (A–D), is 1.34 mm, corresponding to a 4.41% difference. Further analysis reveals that the average change in diameter for inclusion 1, calculated by averaging the changes in all four cases, is 0.35 mm, which represents a 3.56% difference. For inclusion 2, the mean change is determined by averaging the results from phantom C and D, yielding a value of 0.74 mm or a 12.71% difference. Regarding the spatial accuracy, the mean centre offset for the phantoms was 0.62 mm across all cases. For inclusion 1, the mean error was 0.85 mm, while inclusion 2 had an error of 1.13 mm. These positional errors are visually depicted in Fig. 9 (a.v), (b.v) and 10 (c.v), (d.v) and quantitatively assessed using the specified metrics, which highlight the accuracy and reliability of the CNN-based approach in reconstructing SoS maps from UCT data.Fig. 11Bar graphs of: (a) Change% in width and (b) spatial centre offset of phantoms and inclusions, between the CNN reconstructed and ground truth images.Fig. 11

**(a):**
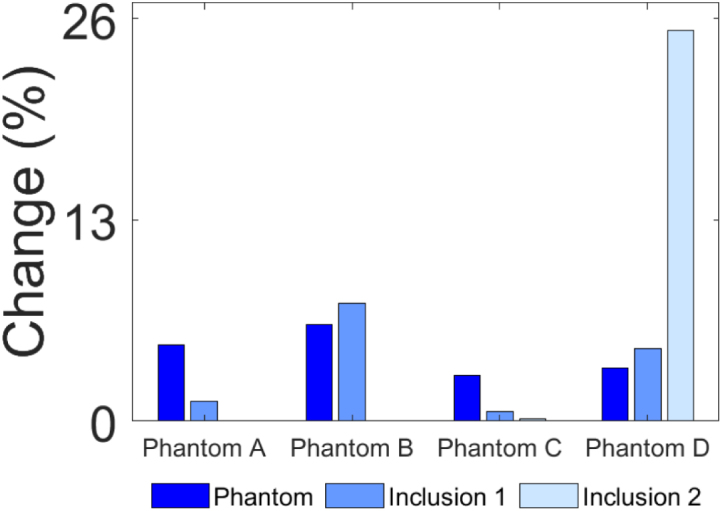
.

**(b):**
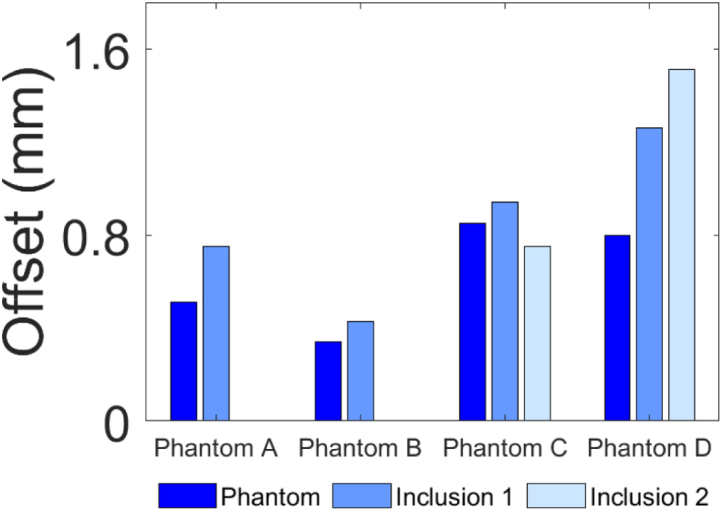
.

## 5 Discussion, limitations and future work

**Fig. E.13:**
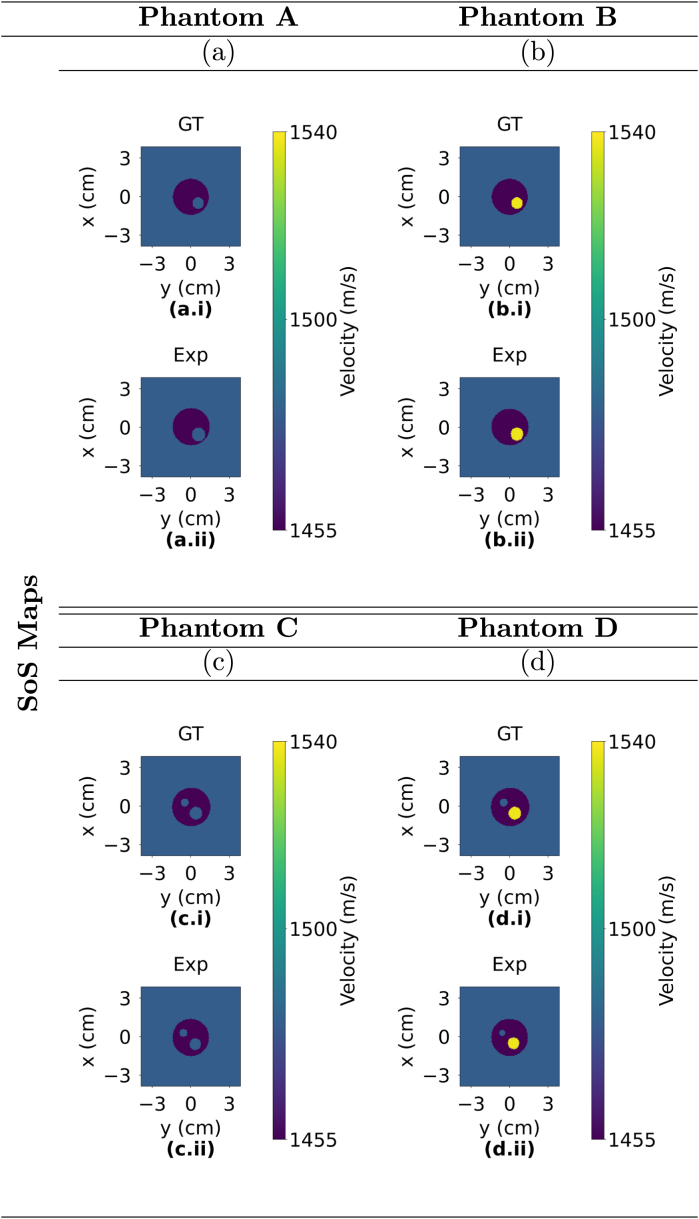
The ground truth SoS maps measured using a camera positioned vertically above the samples and the CNN-predicted SoS maps for all phantoms.

Comparisons between the CNN predicted and ground truth SoS maps are shown in Fig. 9, Fig. 10, Fig. E.13 as well as Table D.4. Overall, the low error values demonstrate that the proposed framework can produce highly accurate SoS reconstructions in terms of sizing, while detecting SoS differences as small as 25 m/s between gel and water, highlighting the system’s sensitivity. The results show that the minimum and maximum sizing error observed is 0.01 mm (Dia. of inclusion 2 in phantom C) and 1.91 mm (Dia. of phantom B), respectively. Similarly, the minimum and maximum centre offset is 0.34 mm (phantom B) and 1.51 mm (inclusion 2 in phantom D), respectively. Fig. 11(a) shows that the water-filled inclusion 2 of phantom D is approximately 25% smaller than the ground truth. This is likely due to the larger SoS variation in phantom D (83 m/s vs 25 m/s in other phantoms), which may reduce the resolution of the SoS reconstructions. Further investigation is needed to confirm the cause.

In [71], tumour widths were measured using three different imaging modalities (MM, conventional ultrasound and MRI) and compared against the pathology measurements. The mean error between each imaging modality and the pathological reference were calculated and expressed as percentage changes in size, analogous to the Change% employed in the present study. The reported Change% values were 59.11% for MRI, 18.34% for conventional ultrasound and 12.64% for MM. Notably, these errors exceed those observed in the present study, which yielded a Change% of 4.41% for the phantoms and 3.56% for inclusion 1, and were only marginally below the error for inclusion 2, when compared with MM, which was found to be 12.71%. Although these findings suggest an improved measurement accuracy in the presented methodology, a direct controlled comparison is needed to assess mean errors between imaging modalities. Future studies should use phantoms imaged under identical conditions with all four techniques.

Biomedical applications are expected to involve a broader range of SoS values and more complex biological structures than those considered in this study. These differences are likely to affect the structures and patterns observed in the experimental ToF matrices, which serve as the CNN input in the presented framework [72]. However, research conducted in [30] demonstrated that CNNs trained on simulated SoS maps, including both simple geometrical models (e.g. ellipses) and complex breast models, were capable of reconstructing accurate SoS distributions in both cases, suggesting robustness of the deep learning approach across different structural complexities. It is also worth noting that extending the training data to encompass arbitrary 3D structures would require an extension of the ALI-FMM [44] to three dimensions. Although this has not yet been addressed in the literature, the ALI-FMM belongs to the broader family of fast marching methods, which have been routinely extended to handle wave front propagation in 3D [73].

The current limitations with respect to the minimum detectable SoS difference and the inclusion size may be further mitigated by broadening the parameter distribution and increasing the size of the training dataset. This has been shown to increase the generalisability and accuracy of the model [41]. Additionally, more accurately simulated ToF data that more closely approximates experimentally acquired data could be obtained by applying a finer grid size in ALI-FMM algorithm [44]. This could further improve the performance of the trained CNN, improving its sensitivity to smaller differences in SoS and finer spatial features. However, further analysis is required to determine the minimum SoS and spatial variations that the network can reliably detect.

The CNN demonstrated high computational efficiency, reconstructing a single SoS map from ToF data within 5.84 ms. In contrast, traditional iterative methods, such as full-waveform inversion (FWI), which are typically employed for reconstruction of SoS maps from UCT data, often require several minutes to hours depending on the experimental parameters [30], [68], [74], [75]. In addition, the study in [30], provides a comprehensive comparison between deep learning-based tomography approaches and classical iterative methods, including straight-ray and bent-ray Simultaneous Algebraic Reconstruction Technique (SART). Their results demonstrate that deep learning models trained on travel-time data consistently outperform both straight-ray and bent-ray SART across key quantitative metrics, while enabling reconstruction speeds of up to three orders of magnitude faster.

Although the focus of the presented study was on tomographic reconstruction speed, a special note is needed regarding the data acquisition speed. The total acquisition time for the two datasets ($T_{1}$ and $T_{2}$) captured for each phantom, was approximately 28 min, due to data acquisition constraints associated with the available oscilloscope. However, this acquisition time can be substantially reduced by utilising an experimental setup optimised for speed, such as the one presented in [76], which achieves higher data transfer rates and employs a galvanometer mirror system for steering the laser ultrasonic generation beam. It is estimated that such a system could acquire a full dataset ($T_{1}$ and $T_{2}$) in approximately 2 min and 20 s. The data acquisition time can be further reduced through the use of spatially encoded ultrasonic generation [77], [78], which reduces signal-independent noise by averaging multiple signals in parallel, thus improving the SNR and reducing the need for signal averaging.

The use of the ALI-FMM algorithm enabled the automated creation of 20,000 unique pairs of ToF matrices and corresponding SoS maps with various geometries and material properties. Although the training dataset was generated off-line, it took approximately 110 h to generate it, while the training process itself was 10 h and 15 min using the PC with the specifications outlined in Section 2.3. These durations are expected to increase if certain array configuration parameters are modified such as the addition of ultrasonic array elements or if a higher image resolution is desired, thereby imposing computational constraints that may hinder scalability. These computational constraints could be mitigated, however, through the use of high-performance computing resources such as a supercomputer with greater processing capabilities than the PC used in this study.

It is worth noting that the CNN used in this study was trained only once and subsequently used to reconstruct the SoS maps for the experimentally acquired data from all phantoms, without the need for retraining. Therefore, if further experimental data were acquired, they could be reconstructed with the same CNN, provided that the same experimental array configuration (array geometry and number of elements) was used. The trained CNN could also be further improved by the creation of additional training data, incorporating more complex geometries and material properties. By allowing for a continuously expanding database of training data, the CNN could then be progressively trained, expanding its usability and improving its accuracy over time. This would significantly reduce the time needed to update the CNN as well as having previously simulated data that could be reutilised for subsequent training cycles. Additionally, transfer learning could also be applied to the existing CNN to accommodate new simulated and experimental datasets [79]. However, new training data would be required and subsequent retraining of the CNN if the configuration parameters of the two ultrasonic arrays were to change (e.g. different element distribution or number of elements).

**Table D.4:** Comparison of phantom and inclusion dimensions between experiment-based predictions of the CNN (Exp) and the ground truth (GT). The diameter of the phantom and inclusions (Dia.), the change in diameter between the ground truth and the CNN predicted image (Change=GT−Exp) and the difference between the ground truth and the CNN predicted image expressed as a percentage difference (Change%) are presented. The location of the centre of the phantom and the inclusions in x and y coordinates (Cent. (x, y)) is given with respect to the centre of the immersion tank. The difference between Exp and GT of these centre locations is also presented.

Phantom A		Phantom			
		GT (mm)	Exp (mm)	Change (mm)	Change (%)
	Dia.	30.5	28.99	1.51	4.95
	Cent. (x, y)	(0.08, 0.25)	(0.55, 0.05)	(−0.47, 0.20)	
		Inclusion			
	Dia.	10.00	9.87	0.13	1.30
	Cent. (x, y)	(−5.83, 5.92)	(−5.25, 5.44)	(−0.58, 0.48)	
Phantom B		Phantom			
		GT (mm)	Exp (mm)	Change (mm)	Change (%)

	Dia.	30.50	28.59	1.91	6.26

	Cent. (x, y)	(0.08, 0.25)	(0.31, 0.00)	(−0.23, 0.25)	

	Inclusion			

Dia.	10.00	9.24	0.76	7.60

Cent. (x, y)	(−5.83, 5.92)	(−5.47, 5.69)	(−0.36, 0.23)	

Phantom C		Phantom			
		GT (mm)	Exp (mm)	Change (mm)	Change (%)

	Dia.	30.02	29.13	0.89	2.96

	Cent. (x, y)	(−0.65, 0.24)	(−0.43, −0.58)	(−0.22, 0.82)	

		Inclusion 1			

	Dia.	9.36	9.30	0.06	0.64

Cent. (x, y)	(−5.57, 3.95)	(−5.73, 3.02)	(0.16, 0.93)	

	Inclusion 2			

Dia.	5.82	5.81	0.01	0.17

Cent. (x, y)	(2.91, −5.00)	(2.95, −5.75)	(−0.04, 0.75)	

Phantom D		Phantom			
		GT (mm)	Exp (mm)	Change (mm)	Change (%)

	Dia.	30.02	28.98	1.04	3.46

	Cent. (x, y)	(−0.65, 0.24)	(−0.33, −0.49)	(−0.32, 0.73)	

		Inclusion 1			

	Dia.	9.36	8.92	0.44	4.70

Cent. (x, y)	(−5.57, 3.95)	(−5.15, 2.76)	(−0.42, 1.19)	

	Inclusion 2			

Dia.	5.82	4.35	1.47	25.26

Cent. (x, y)	(2.91, −5.00)	(3.78, −6.24)	(−0.87, 1.24)	

Given that decreasing the width of the laser line source leads to increasingly divergent acoustic signals [80], and that the ablation threshold rises with higher PDMS to CS ratios [16], further investigation of both parameters is required. The goal of this optimisation is to generate ultrasonic pulses with sufficient amplitude and directivity at all source locations, ensuring accurate ToF picking across all detection points, particularly in highly attenuating or thicker phantoms. At the same time, these parameters must be balanced to prevent coating ablation, thereby enabling repeated use of the generation plate.

Finally, the validity of the proposed two-stage ToF picker method was evaluated by comparing its ToF selections with both single-stage kurtosis and single-stage cross-correlation selections against manually verified ToFs, which served as the ground truth, for one ToF matrix. Quantitative analysis revealed that the single-stage kurtosis-based method (where the first kurtosis peak exceeding a user-defined threshold of 55% was selected as the ToF) yielded a mean difference of 19.234 ns and a standard deviation of the difference of 19.265 ns while the single-stage cross-correlation method yielded a mean difference of 64.081 ns and a standard deviation of 141.401 ns. The proposed two-stage method demonstrated a lower mean difference of 6.191 ns relative to the manually verified ToF, compared to either of the single-stage methods and a standard deviation of 30.237 ns, which is slightly higher than that of the single-stage kurtosis but lower than that of the single-stage cross-correlation method. Overall, the addition of the second stage enhanced ToF detection accuracy, highlighting its effectiveness over single stage techniques, which lacked the consistency required to select the true ToF.

## 6 Conclusions

The aim of this study was to investigate the feasibility of reconstructing SoS maps of ultrasound tissue mimic phantoms in near-real time using a CNN applied to LU data. The trained CNN used experimental ToF matrices as input and produced SoS maps of the investigated medium. The reconstructed SoS maps exhibited a high degree of accuracy in SoS distribution, as well as the size and location of the phantom and inclusions, when compared to ground truth data. These results provide quantitative validation of the CNN’s ability to reconstruct the SoS maps that closely align with the ground truth. Although the CNN was trained exclusively on synthetic data generated by a physics-based model (no experimental data were used), it still achieved accurate SoS map reconstructions when tested on experimental data. Furthermore, the reconstruction process was completed in <6 ms, demonstrating the potential of DL for real-time tomographic reconstructions, offering a fast alternative to traditional, iterative, ToF tomography methods.

This work demonstrates the potential of CNNs for tomography reconstruction of tissue-mimicking phantoms using data captured by an all-optical setup based on laser ultrasonics. The system was tested with four phantom variations to evaluate its effectiveness. Results indicated that the similarity between the CNN predictions and their corresponding ground truth SoS maps was superior to that achieved by other imaging modalities, including MM, conventional ultrasound and MRI, based on results from previously published literature. This highlights the potential for this method to be used for biomedical imaging applications that require precise tissue differentiation based on acoustic properties. Overall, this study demonstrates that the integration of deep learning with LU has significant potential for advancing non-invasive biomedical imaging, offering both real-time capability and enhanced diagnostic precision.

## CRediT authorship contribution statement

**Ahmed Al Fuwaires:** Writing – review & editing, Writing – original draft, Visualization, Validation, Software, Investigation, Formal analysis, Data curation. **Peter Lukacs:** Supervision, Methodology, Data curation. **Don Pieris:** Investigation. **Geo Davis:** Investigation. **Helen Mulvana:** Writing – review & editing, Writing – original draft, Supervision, Methodology, Funding acquisition, Formal analysis, Conceptualization. **Katherine Tant:** Writing – review & editing, Writing – original draft, Supervision, Software, Methodology, Formal analysis, Conceptualization. **Theodosia Stratoudaki:** Writing – review & editing, Writing – original draft, Supervision, Resources, Project administration, Methodology, Funding acquisition, Formal analysis, Conceptualization.

## Figures and Tables

**Fig. C.12:**
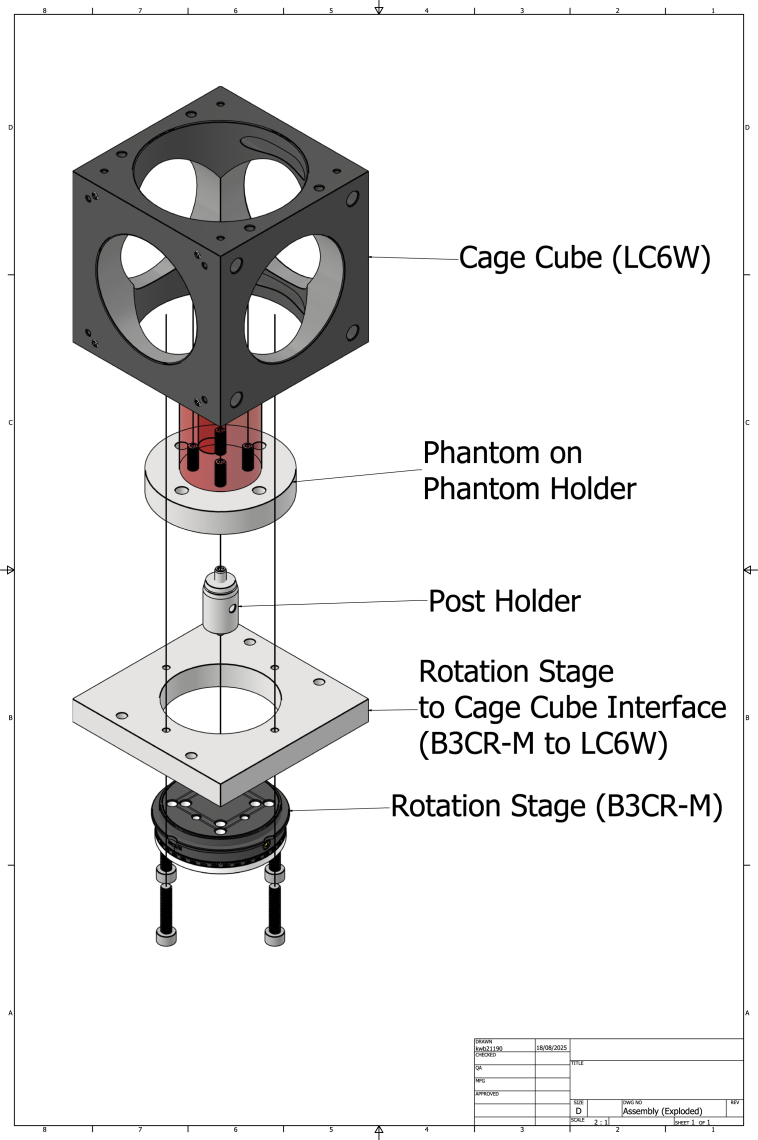


## Data Availability

data on public repository. Link has been attached. University of Strathclyde KnowledgeBaseData for: ”Neural networks for faster laser ultrasound tomography in tissue phantoms” (Original data) .

## Appendix A CSNP fabrication

CSNPs were produced through the flame synthesis process reported in [14] until a total of 125 mg of candle soot (CS), the amount required to coat a circular glass plate 50.8 mm in diameter, was synthesised and retained in a container. The PDMS base and curing liquid (Sylgard 184, Sigma Aldrich) was mixed at a ratio of 10:1 and centrifuged at 1300 RPM for 2 min to ensure a homogeneous mixture and remove bubbles. CS was then added to the PDMS at a ratio of 5:1 (PDMS:CS) based on the process described in the literature [81]. The mixture was centrifuged at 1300 RPM for 10 min to ensure uniformity then spin coated on a glass plate (WG12012, Thorlabs) at 1000 RPM for 100 s, then 1500 RPM for 800 s and finally 2500 RPM for 400 s to ensure a thin, uniform coating. The coated glass plate was subsequently placed in an oven at 90 °C for 60 min to cure.

## Appendix B Phantom fabrication

A custom-made metallic base was attached to a PTFE pipe used as a mould with an inner radius of 30 mm and outer radius of 80 mm. This was then filled with the solid gel and dye (Amber Tone Dye, Humimic Medical) at a ratio of approximately 3:1 (Gel:Dye) and placed in the oven at 125 °C for 2 h until the gel and dye melted to a liquid at which point either one or two metal rods were placed within the mould to create the inclusions. The mixture was placed back in the oven for another 2 h to remove any bubbles created. The gel was allowed to cool for 12 h before being removed from the mould.

## Appendix C Immersion tank setup

See Fig. C.12.

## Appendix D Phantom and inclusion dimensions and measurements

See Table D.4.

## Appendix E SoS maps

See Fig. E.13.
